# An accessible path to nature: piloting virtual nature interventions among adults with disabilities by examining heart rate variability

**DOI:** 10.3389/fpsyg.2026.1851869

**Published:** 2026-09-03

**Authors:** Naike Dorothea Gorr, Ayumu Ochi, Martta Niemi, Minna Huotilainen

**Affiliations:** 1 Faculty of Educational Sciences, University of Helsinki, Helsinki, Finland; 2 School of Management, University of Vaasa, Vaasa, Finland; 3 Centre of Excellence in Music, Mind, Body, and Brain, Faculty of Educational Sciences, University of Helsinki, Helsinki, Finland; 4 EDUCA Flagship, Faculty of Educational Sciences, University of Helsinki, Helsinki, Finland

**Keywords:** adults with disabilities, cardiovascular relaxation, cognitive engagement, heart rate variability, nature exposure, virtual reality

## Abstract

Nature exposure is known to support psychological and physiological well-being, yet individuals living in institutional care often face significant barriers to accessing nature. This pilot study examined the feasibility as well as the physiological effects of tailored virtual nature-based interventions delivered via an immersive dome environment among adults with intellectual and/or physical disabilities. Employing a repeated-measures, within-subject design, this study analyzed heart rate and heart rate variability across multiple interventions to assess physiological responses to virtual nature exposure. Across participants, the interventions were associated with consistent decreases in heart rate variability, while no uniform response pattern was observed for heart rate. Case-level analyses suggested a two-fold response that might depend on the participants’ baseline arousal and behavioral profiles. Individuals predominantly described as calm and bedbound exhibited increased cognitive engagement, as reflected by decreased heart rate variability and increased heart rate, whereas individuals described as restless or active exhibited decreases in both heart rate and heart rate variability, possibly indicating cognitive engagement paired with cardiovascular relaxation. This lack of uniform physiological response suggests that the impact of virtual nature exposure may depend on individual characteristics and contextual needs. Taken together, these findings offer preliminary evidence for the feasibility of virtual nature-based interventions in institutional care settings and point to their potential as an accessible and adaptable approach to supporting stress regulation and cognitive engagement.

## 1 Introduction

Research consistently shows that spending time in nature positively impacts our psychological and physiological well-being. While even brief nature exposure can cause a significant decline in stress markers, long-term exposure is associated with sustained improvements in recovery and stress regulation (e.g., Bratman et al., 2015; Sonntag-Öström et al., 2015; Martin et al., 2020; Roberts et al., 2020). These findings have been replicated with various populations, typically employing interviews, questionnaires, and physiological markers.

Three key theoretical frameworks have been proposed to explain these restorative effects. Wilson’s (1984) biophilia hypothesis suggests that humans are evolutionarily predisposed to search for and find comfort in natural settings; an instinct thought to have evolved to promote survival through the selection of resource-rich and low-risk environments. In line with this theory, the attention restoration theory (ART) states that exposure to natural environments, or even viewing natural landscapes, evokes soft fascination, shifting to effortless attention and thereby facilitating the recovery of directed attention capacities (Kaplan, 1995; Berman et al., 2008). Similarly, Ulrich’s stress reduction theory (Ulrich et al., 1991) proposes that nature exposure, more so than urban settings, evokes positive emotions and physiological relaxation. Together, these theories provide a coherent evolutionary and psychological framework for understanding the benefits of nature exposure, highlighting its potential as an intervention for stress-related symptoms and disorders.

However, it should be considered that not all individuals can easily access natural environments. Individuals with limited mobility or those living in institutional care often face significant barriers when it comes to accessing nature. For these groups, the benefits of nature are mainly inaccessible in daily life. As a response, virtual reality (VR) has emerged as an alternative to deliver virtual nature experiences. Here, the use of VR glasses, immersive room environments, and screens to depict natural scenes has been shown to reproduce many of the stress-reducing and restorative benefits of real nature (Annerstedt et al., 2013; Browning et al., 2020; Li et al., 2021; Kumpulainen et al., 2024). Despite these promising findings, it is important to note that most research has focused on adults without disabilities, leaving individuals with disability under-researched. One group that might particularly benefit from virtual nature exposure is residents of long-term care facilities, as they often have challenges in stress reduction, mobility, and communication. Yet, empirical evidence regarding the feasibility and effectiveness of virtual nature interventions on this population remains scarce.

The present pilot study addressed this gap by examining the effectiveness of tailored virtual nature interventions on residents of a long-term care facility for adults with disabilities in Finland. Unlike previous studies, this study comprised participants with intellectual and/or physical disability. To investigate the potential physiological effect, this study examined heart rate (HR) and heart rate variability (HRV) as indicators for autonomic nervous system functioning (Shaffer and Ginsberg, 2017). This decision was based on their objective nature as measures for stress and vigilance, combined with their suitability for individuals with limited verbal communication skills.

In general, HRV represents the variation in time between adjacent heartbeats and reflects the heart’s ability to respond to stressors in the environment and its recovery (McCraty and Shaffer, 2015). It is considered a common physiological measure in research on stress, vigilance, and mental well-being (Thayer et al., 2012; Kim et al., 2018) and has been used to examine the effect of VR nature interventions (e.g., Kumpulainen et al., 2024). While there are several HRV indicators, the root mean square of successive differences (RMSSD) was chosen in this study due to its reliability in indicating parasympathetic activity and recovery. While RMSSD tends to be more sensitive to cognitive load and stress, HR represents a stable cardiovascular measure, being more sensitive to physical movement (Delliaux et al., 2019).

We hypothesized that the virtual nature interventions would be associated with measurable changes in participants’ HRV and HR relative to their daily averages, and that the direction and magnitude of these changes would depend on each participant’s cognitive and physical profile.

## 2 Materials and methods

### 2.1 Study design

A repeated-measures, within-subject design was employed to evaluate the physiological responses to virtual nature-based interventions over a period of time. The study was primarily conducted in autumn 2024 as part of the NATUREACH development project, which piloted tailored virtual nature service models in five care settings across Sweden and Finland. For this sub-study, data from residents with physical and/or intellectual disabilities of one long-term care facility in Finland were included. This decision was made due to the residents’ limited mobility and minimal access to natural outdoor environments. The virtual nature interventions were conducted using an immersive VR dome tailored for this purpose and the specific user group. The visual and auditory environments were designed and produced based on staff evaluations and participants’ experiences to suit their needs and preferences.

### 2.2 Participants

In total, *N* = 7 adults (sex assigned at birth: 29% female, 71% male; age: *mean* = 37.57 ± 13.01 years, range 22–54 years) residing in a 24/7 residential facility providing specialized care to individuals with physical and/or intellectual disability contributed to the study and data analysis. While the recruitment goal was 10–15 participants, 8 residents initially participated, of whom 7 met the inclusion criteria of having participated in two or more intervention sessions combined with available HRV data.

Participants were recruited with the assistance of the professional nursing staff, who are familiar with each individual’s preferred communication methods. All participants received continuous support in daily living activities, and their mobility arrangements, as well as behavioral symptoms varied (see Table 1). Communication was facilitated through individualized methods such as non-verbal cues, symbols, pictures, and gestures. The facility’s professional nursing staff played a key role in facilitating communication and participation, ensuring that the interventions were person-centered and ethically administered.

**TABLE 1:** Participants’ profiles outlining their mobility arrangements and behavioral symptoms.

Participant ID	Mobility arrangements	Behavioral symptoms
EC01	Bedbound	Mostly calm, attentive in dome
EC02	Bedbound	Mostly calm, attentive in dome
EC03	Bedbound	Mostly calm, attentive in dome
EC04	Uses wheelchair	Active during the day, attentive in dome
EC05	Uses wheelchair	Occasional fearfulness, attentive in dome
EC34	No mobility device	Often restless and agitated, calms in dome
EC36	No mobility device	Often restless and agitated, calms in dome

### 2.3 Ethical approval and consent

The study was approved by the University of Helsinki Ethical Review Board in Humanities and Social and Behavioral Sciences (Statement 14/2024) and conducted in accordance with institutional guidelines. Participation in the study was entirely voluntary. Consent procedures were tailored to each participant’s communication preferences, and informed consent was provided through proxies, support tools, or alternative communication methods where appropriate. If, following established ethical procedures, it was assessed that a resident could not provide informed consent for physiological monitoring, they were not enrolled in that portion of the study. Participation could be discontinued at any time without consequences.

### 2.4 Virtual nature-based intervention

**FIGURE 1:**
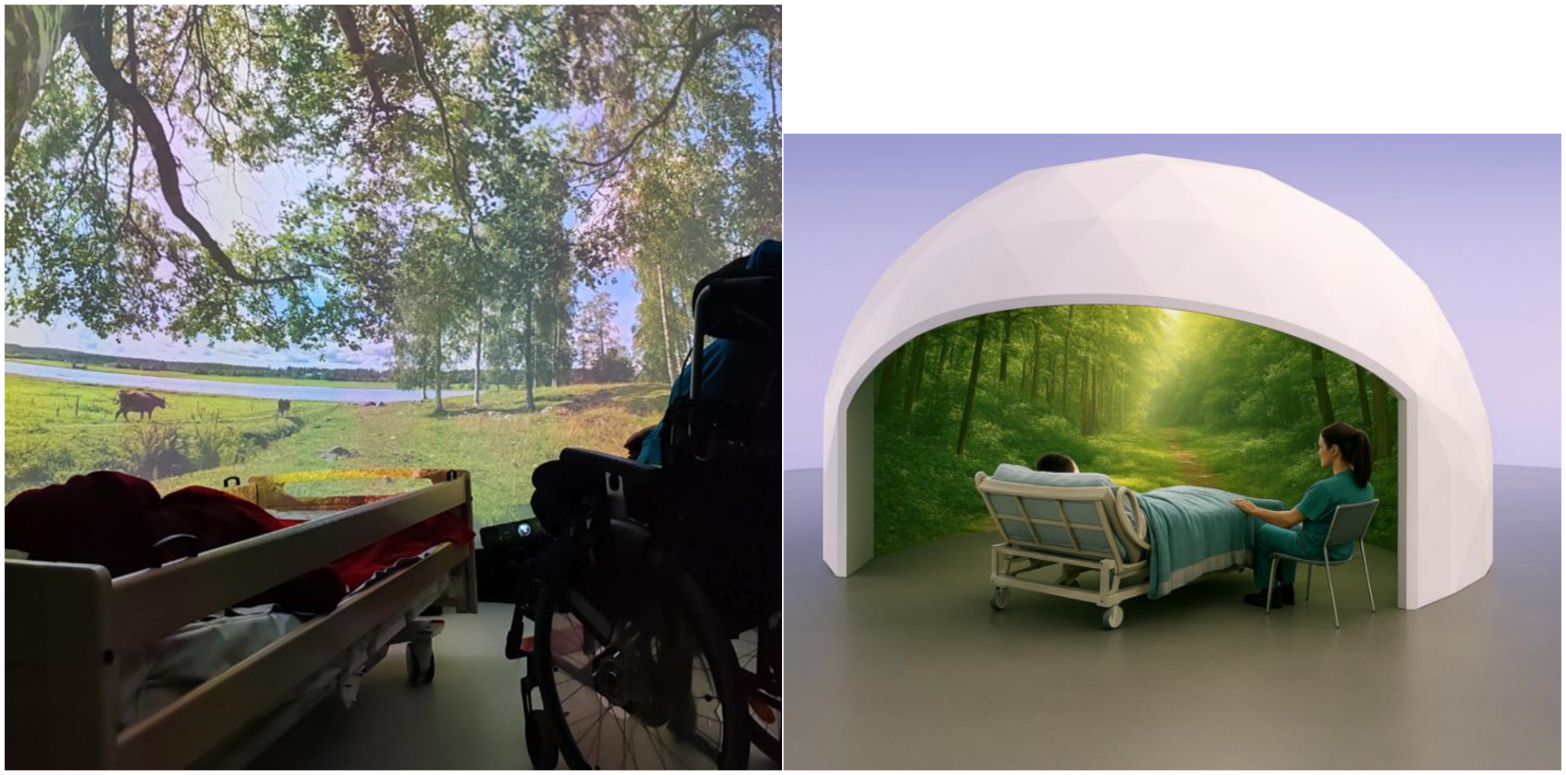
Internal (left) and external (right) image of the dome environment tailored by Polidomes (2023).

The interventions took place in a custom-built dome environment which was installed in the care facility (see Figure 1). The dome consisted of a hemispherical screen with 180-degree video projection covering both walls and ceiling, providing a fully enveloping visual and auditory experience. The dome environment was designed to accommodate participants in wheelchairs or hospital beds, ensuring physical accessibility.

The high-resolution panoramic nature videos depicted various calming scenes (e.g., forests, lakesides, open meadows) but also natural environments with moving elements such as grazing cows and lambs or moving in a boat along a river. Each video lasted about 15 min, and participants could freely choose both the type of video and the viewing time, with the assistance of a care professional. On average, the sessions lasted between 30 and 60 min, depending on the participant’s preferences and comfort level. Sessions were repeated over an average of 7 weeks (range: 4–11 weeks), with frequency ranging from zero to four sessions per week. The participants’ dome visits, session length, and chosen video environment were recorded in diaries maintained by the professional nursing staff.

### 2.5 Physiological data collection

Physiological responses were collected using the Firstbeat Bodyguard2 device, which records consecutive heartbeats (R-R intervals) and movement data. The compact device was attached to the participant’s upper body using two adhesive electrodes, one below the right collarbone and the other on the left rib cage. The device was worn for up to four consecutive days per session, and only removed for swimming, sauna, showering, or charging. Data collection was initiated approximately 1 day prior to the scheduled dome visit to capture both baseline and post-intervention periods.

During the measurement window, the goal was to capture at least one dome visit per recording. The device was removed earlier if the participant expressed discomfort or no longer wished to wear it. As with all components of the study, participation in physiological monitoring was optional and based on the judgment of both staff and participants regarding understanding, comfort, and voluntariness.

### 2.6 Data handling and cleaning

The obtained Firstbeat data and accompanying diaries were uploaded and stored on secure servers at the University of Vaasa. From there, researchers at the University of Helsinki accessed the data in spring 2025 for data cleaning and diary coding. All data were pseudonymized and handled in compliance with data protection regulations. No personally identifiable information was included in the data analysis or publication. The Firstbeat data were cleaned using a custom procedure developed at the University of Helsinki to minimize noise without excessive data loss (Salonen, manuscript in preparation). Artifact correction of interbeat interval (IBI) data was performed using a heuristic approach. In this procedure, single extra beats and missed beats were modeled to preserve the preceding physiological pattern observed in artifact-free data. All corrected data points were flagged and could be excluded from subsequent analyses if required.

Rather than applying time-based segmentation, heart rate variability (HRV) indices were calculated for each beat in the artifact-corrected IBI series using the Marcus Vollmer HRV Toolbox for MATLAB (Vollmer, 2019). A detailed description of the artifact-cleaning procedure and IBI data handling methods will be published separately (Salonen, manuscript in preparation). Because the dataset does not include raw ECG recordings, it was not possible to verify individual beats or perform beat-level analyses directly from the original physiological signal.

Using the cleaned HRV data, standard HRV parameters were calculated in Microsoft Excel, including the root mean square of successive differences (RMSSD), the standard deviation of NN intervals (SDNN), the standard deviation of successive differences (SDSD), the percentage of successive NN intervals differing by more than 50 ms (pNN50), RR interval variability (RRHV), parasympathetic function level (PFL), parasympathetic nervous function (PNF), very low frequency power (VLF), low frequency power (LF), and the low-frequency to high-frequency power ratio (LF/HF). RMSSD and HR were selected as primary outcome parameters due to both methodological and practical reasons. First, RMSSD was selected due to its reliability even with short-duration recordings, not requiring stable, longer measurement windows. Given the variation in intervention durations both within and across participants, RMSSD was deemed most suitable as an indicator of parasympathetic activity. Second, HR was selected as a complementary parameter due to its stability and interpretability as a cardiovascular indicator. Its sensitivity toward physical exertion was deemed important given the heterogenous mobility profiles of our participants. Measurements were time-aligned with dome session logs to identify the intervention windows.

### 2.7 Statistical analysis

In order to examine the potential impact of virtual nature-based interventions on the participants’ HR and HRV, we compared the mean scores during each intervention with the mean scores of that same day. By comparing the intervention values with their daily average instead of the participant’s overall average, we obtained a more contextually sensitive baseline accounting for daily factors such as sleep quality, illness, and mood. The adjacent time windows were considered as an alternative comparison but were rejected on the grounds that physically relocating the participants to and from the dome would likely lead to measurable physiological responses, rendering those time windows unreliable as a baseline.

The daily mean was computed by calculating the mean values between 9 a.m. and 10 p.m., excluding the intervention window. The intervention mean itself was computed by calculating the mean of the final 25 min of each intervention session, providing us with comparable intervention windows. For example, if the intervention lasted 30 min, we excluded the first 5 min for stabilization and then included the final 25 min in the analysis. In case of missing end times, applicable to four sessions originating from EC01, EC02, EC03, and EC04, we assumed that the intervention lasted 30 min and proceeded with the same steps. Interventions lasting less than 30 min were still included in the analysis, given that they exceeded 10 min, allowing for a 5-min stabilization window as well as a 5-min window for determining the scores.

Finally, we conducted two paired-samples *t*-tests to determine whether virtual nature interventions had a statistically significant impact on the participants’ RMSSD and HR scores. This analysis was deemed appropriate given that each intervention average was directly paired with the daily average, reflecting a paired comparison. A linear mixed-effects model, which would account for the nested structure of the repeated sessions within participants, was initially considered, however, the small sample size (*N* = 7) rendered this analysis unfeasible. Paired-samples *t*-tests were therefore selected as a suitable alternative given the constraints of this pilot study. Prior to analysis, the daily and intervention averages, as well as their paired differences, were examined for normality and outliers. While normality was assessed using Shapiro-Wilk tests and visual inspection of histograms, outliers were identified through visual inspection of the paired difference-score distributions. The examination revealed slight deviations from normality in the RMSSD and HR data, and one potential outlier in the RMSSD data. Following the exclusion of the potential outlier, RMSSD difference scores did not show significant deviations from normality, but HR difference scores deviated from normality. Given the sample size, paired-samples *t*-tests were retained as primary analysis for both outcomes due to their relative robustness to violations of normality. To further verify the robustness of the results, two sensitivity analyses were conducted for each outcome. The primary analyses were based on *N* = 45 sessions for RMSSD and *N* = 46 sessions for HR.

The *t*-tests as well as accompanying visualizations were conducted using IBM SPSS Statistics 29.0 (IBM Corporation, Armonk, NY, USA).

## 3 Results

### 3.1 Results of descriptive statistics

#### 3.1.1 Overview of interventions

On average, the participants (*N* = 7) completed 13 dome sessions (range: 8–21) across an average intervention period of 7 weeks (range: 4–11 weeks; see Table 2). The mean session duration was 38.5 min (range: 15–120 min). After applying the inclusion criteria, several sessions were excluded from the statistical analysis due to missing HRV data, missing timestamps, or technical error with the Firstbeat device (see Table 2). One session from EC34 was excluded from the statistical analysis as the recorded heart rate exceeded 209 bpm and hence was identified as physiologically impossible and regarded as a technical error of the recording device. Finally, data collected after spring 2025 were not included in the present analysis. While the data collection was ongoing until autumn 2025 as part of the wider project, a manuscript cut-off date was set to ensure a timely manuscript preparation to meet the publication deadline.

**TABLE 2:** Participant-level overview of interventions.

ID	Total # interventions	Intervention period (weeks)	Interventions per week	Total min watched	$\bar{\text{X}}$ session length (min)	Excluded interventions (reason)
EC01	16	9	2	760	47.5	9 (2 missed HRV data; 7 recorded in summer 2025)
EC02	21	11	2	851	40.5	7 (1 missed HRV data; 6 recorded in summer 2025)
EC03	11	9	1	360	33	9 (2 missed HRV data; 3 missed start/end time; 4 recorded in summer 2025)
EC04	9	8	1	345	38.5	6 (1 missed HRV data; 5 recorded in summer 2025)
EC05	11	5	2	480	43.5	1 (1 missed HRV data)
EC34	12	4	3	355	29.5	5 (1 missed HRV data; 4 excluded due to technical error)
EC36	8	4	2	237	29.5	5 (excluded due to technical error)

#### 3.1.2 Session-level data

**FIGURE 2:**
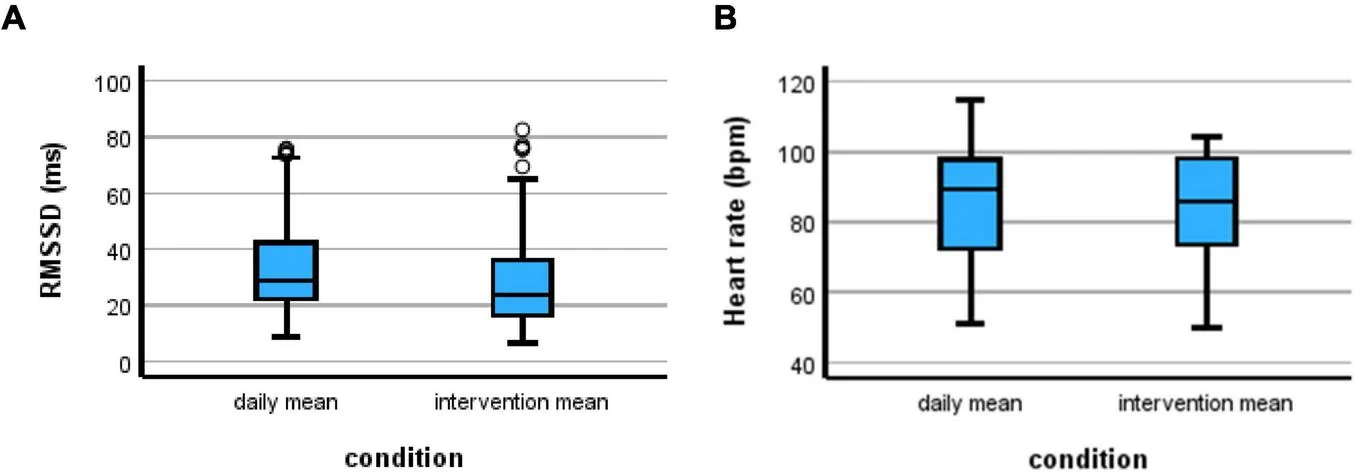
**(A)** Mean RMSSD scores across participants. **(B)** Mean HR scores across participants. The middle line of the box represents the median while the bottom and top lines of the box represent the interquartile range. The vertical lines extending from the ends of the box indicate variability outside the upper and lower quartiles.

Across participants, *mean* RMSSD scores were lower during intervention sessions compared to their daily averages (see Figure 2A). Specifically, daily *mean* RMSSD decreased from 36.56 ms (*N* = 46, min = 8.8, max = 75.9, SD = 20.24) to 31.00 ms during intervention (*N* = 46, min = 6.2, max = 82.6, SD = 21.59). This trend was also visible on the individual level, with all participants exhibiting lower average RMSSD scores during the intervention relative to their daily average (see Table 3).

**TABLE 3:** Participants’ RMSSD and HR changes across the intervention period.

ID	Distribution of intervention time	HRV, HR measure	Interventions included	Interventions with decline	Interventions with increase	Daily averages	Intervention averages	Centralized scores	Std. deviation of centralized scores
EC01	Morning: 3 Afternoon: 1 Evening: 3	RMSSD	7	5	2	30.2 ms	26.1 ms	−4.10	5.80
		HR	7	2	5	88.4 bpm	92.9 bpm	4.42	6.56
EC02	Morning: 3 Afternoon: 10 Evening: 1	RMSSD	14	6	8	50.6 ms	48.5 ms	−2.12	9.93
		HR	14	4	10	71.9 bpm	74.3 bpm	2.35	4.75
EC03	Morning: 1 Afternoon: 1 Evening: 0	RMSSD	2	2	0	47.6 ms	40.4 ms	−7.25	2.97
		HR	2	1	1	79.0 bpm	81.2 bpm	2.12	3.34
EC04	Morning: 1 Afternoon: 2 Evening: 0	RMSSD	3	2	1	53.9 ms	47.1 ms	−6.78	9.87
		HR	3	3	0	60.2 bpm	56.8 bpm	−3.38	2.79
EC05	Morning: 2 Afternoon: 5 Evening: 3	RMSSD	10	8	2	18.7 ms	14.6 ms	−4.12	5.68
		HR	10	4	6	96.9 bpm	97.5 bpm	.55	4.20
EC34	Morning: 2 Afternoon: 5 Evening: 0	RMSSD	7	6	1	35.8 ms	20.4 ms	−15.40	21.89
		HR	7	5	2	105.3 bpm	94.0 bpm	−11.33	11.72
EC36	Morning: 2 Afternoon: 1 Evening: 0	RMSSD	3	3	0	22.5 ms	18.0 ms	−4.41	1.74
		HR	3	3	0	77.3 bpm	75.1 bpm	−2.21	1.61

Intervention times were classified into morning sessions (between 9 a.m. and 12 p.m.), afternoon sessions (between 12 p.m. and 5 p.m.), and evening sessions (between 5 p.m. and 10 p.m.).

In contrast, heart rate (HR) values did not show noticeable changes when comparing intervention sessions (*N* = 46, *M* = 84.4, SD = 14.67, min = 49.8, max = 104.3) to their daily averages (*N* = 46, *M* = 84.9, SD = 15.63, min = 51.0, max = 115.0) (see Figure 2B). On the individual level, responses were mixed showing a decline in HR for EC04, EC34, and EC36, whereas the remaining participants exhibited an increase in HR during the intervention (see Table 3).

#### 3.1.3 Time series data

**FIGURE 3:**
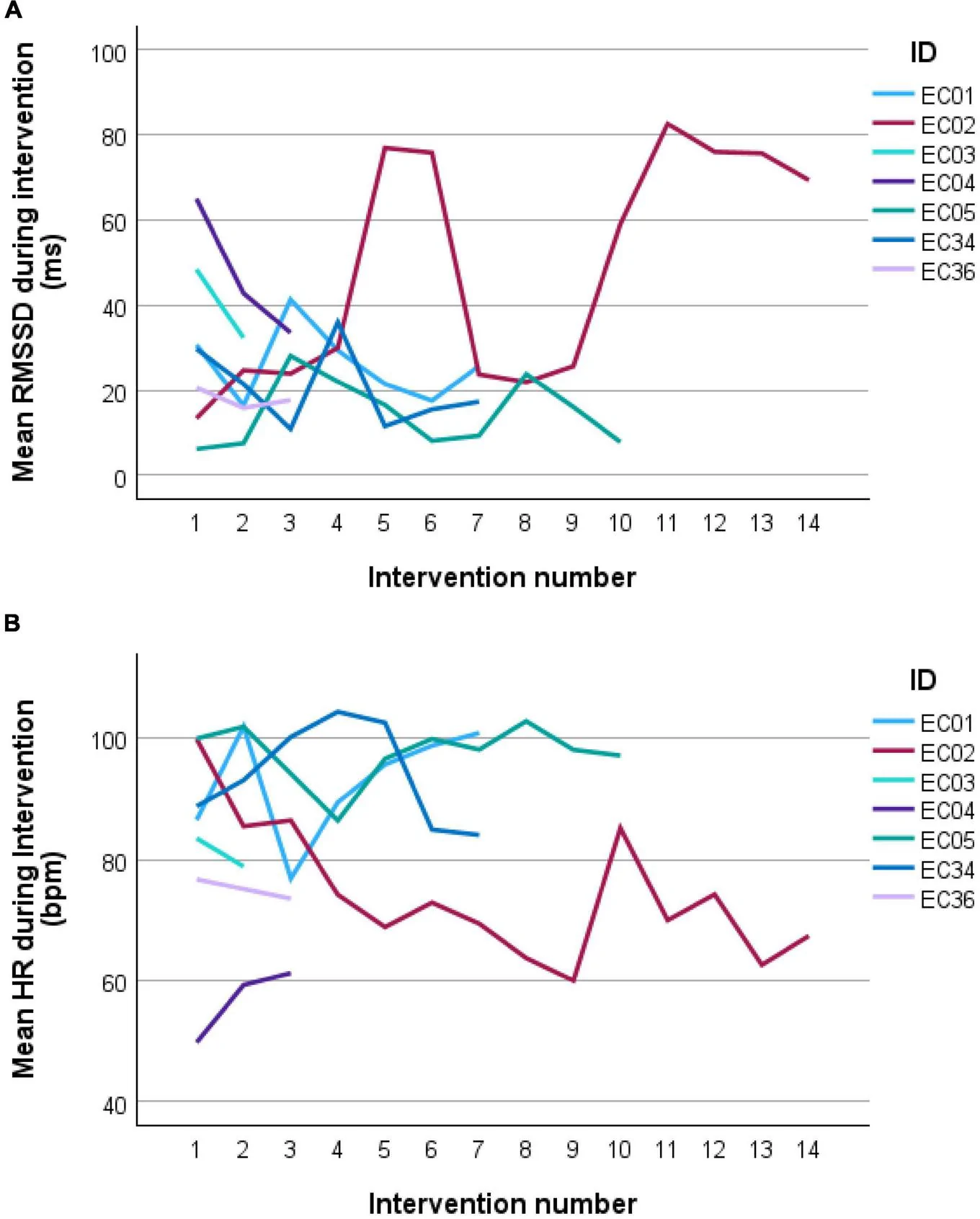
**(A)** Time-series plot showing the participants’ *mean* RMSSD across intervention sessions over time. **(B)** Time-series plot showing the participants’ *mean* HR across intervention sessions over time.

Shifting from the session level to the time series data revealed considerable variation both within and across participants, with no apparent cumulative effect over time for either RMSSD or HR (see Figures 3A, B).

### 3.2 Results of statistical analysis

Examination of the daily and intervention average scores revealed non-normal distributions for the daily and intervention average RMSSD scores and the intervention average HR scores, whereas the daily average HR scores did not significantly deviate from normality. For RMSSD, visual inspection of the histogram led to the identification of one potential outlier originating from EC34, revealing a difference score of 58.07 ms, larger than the other difference scores for EC34 (17.87, 16.97, 15.56, 11.87, 9.31, and 9.27 ms). A subsequent Shapiro-Wilk test revealed that the exclusion of the outlier led to a clear improvement in normality (prior exclusion: *W* = 0.84, *p* < 0.001; post exclusion: *W* = 0.98, *p* = 0.628). The primary RMSSD analysis was therefore conducted with 45 paired observations. For HR, visual inspection did not suggest any outliers, leading to the inclusion of the full sample of *N* = 46 sessions. The histogram and Shapiro-Wilk test revealed that the difference scores between the daily average and intervention average of HR deviated from normality (*W* = 0.91, *p* = 0.002). However, given the sample size of 46 paired observations, the paired-samples *t*-test was retained as the primary analysis considering its relative robustness.

The following results were obtained from these analyses. In the context of RMSSD, the primary test showed a statistically significant decrease in RMSSD when comparing the daily average (*M* = 35.69, SD = 19.59) to the intervention average (*M* = 31.31, SD = 21.74), *t*(44) = 3.60, *p* < 0.001 (two-tailed). The mean decrease in RMSSD was 4.39 ms, with a 95% confidence interval ranging from 1.93 to 6.84. The eta squared statistic (η^2^ = 0.23) indicated a large effect size (Cohen, 1988, pp. 284–287; 0.01 = small effect, 0.06 = moderate effect, 0.14 = large effect). To verify the robustness of the findings, two sensitivity analyses were conducted. First, the paired-samples *t*-test was repeated including the excluded outlier originating from EC34 (*N* = 46) yielding a comparable result with a significant decrease from daily average (*M* = 36.56, SD = 20.24) to intervention average (*M* = 31.00, SD = 21.59), *t*(45) = 3.33, *p* = 0.002 (two-tailed). The mean decrease in RMSSD was 5.55 ms, with a 95% confidence interval ranging from 2.20 to 8.91. The eta squared statistic (η^2^ = 0.20) indicated a large effect size (Cohen, 1988). Second, given that the nested structure of the data violated the independence assumption of the paired-samples *t*-test, a further sensitivity analysis was conducted by aggregating the intervention scores to participant-level means (*N* = 7). The result again supported the statistically significant decrease in RMSSD from daily average (*M* = 37.04, SD = 14.01) to intervention average (*M* = 30.72, SD = 14.31), *t*(6) = 3.82, *p* = 0.009 (two-tailed), with a mean decrease of 6.31 ms (95% CI: 2.27–10.35) and a large effect size (η^2^ = 0.71; Cohen, 1988). Taken together, both sensitivity analyses support the robustness of the primary findings.

In the context of HR, the daily average (*M* = 84.85, SD = 15.63) did not differ significantly from the intervention average (*M* = 84.37, SD = 14.67), *t*(45) = 0.42, *p* = 0.673 (two-tailed). The mean decrease in HR was 0.49 bpm, with a 95% confidence interval ranging from −1.83 to 2.80. The eta squared statistic (η^2^ = 0.004) indicated a negligible effect size (Cohen, 1988). Two sensitivity analyses were similarly conducted to confirm the result. First, aggregating the intervention scores to participant-level means (*N* = 7) likewise showed no statistically significant change in HR from daily average (*M* = 82.73, SD = 15.34) to intervention average (*M* = 81.67, SD = 14.40), *t*(6) = 0.54, *p* = 0.611 (two-tailed), with a mean decrease of 1.07 bpm (95% CI: −3.81 to 5.94). The eta squared statistic (η^2^ = 0.05) indicated a small effect size (Cohen, 1988). Second, given that the difference in HR scores violated the normality assumption, a Wilcoxon signed-rank test (*N* = 46) was conducted as a further sensitivity analysis, likewise indicating no statistically significant difference between the daily average (*Md* = 89.26) and intervention average (*Md* = 85.93), *z* = 0.399, *p* = 0.690, with a negligible effect size (*r* = 0.04; Cohen, 1988). In the calculation of the effect size, *N* represented the number of total observations over two conditions (*N* = 92). As with the RMSSD analyses, both sensitivity analyses support the robustness of the primary HR findings.

## 4 Discussion

### 4.1 Overview of main findings

Taking a sample-wide perspective, the present pilot study suggests that virtual nature-based interventions were associated with statistically significant reductions in participants’ RMSSD, indicating a decrease in parasympathetic nervous system activity. While no statistically significant changes were found for heart rate, descriptive statistics revealed considerable variability in the direction and magnitude of physiological responses. This variability suggests that virtual nature-based interventions did not evoke uniform autonomic responses and that instead the sample’s heterogeneity (incl., mobility and disability profiles) might have impacted the observed response patterns. This finding suggests that a sample-wide analysis alone is insufficient for populations with significant intra- and interindividual differences. Instead, it requires an additional participant-level analysis to understand how certain contextual factors impact the autonomic response to virtual nature. Below, we expand upon this heterogeneity by summarizing participant-specific responses in the light of contextual and behavioral characteristics as outlined in section “2.2 Participants.” It should be noted that these interpretations remain mostly speculative, being predominantly based on the physiological data alone, supplemented to a small degree by the staff observations and descriptions obtained by the wider project context.

### 4.2 Case-level heterogeneity

#### 4.2.1 EC01

EC01 visited the dome 16 times, with sessions distributed across morning and evening slots and an average duration of 47.5 min. Their profile, namely bedbound, calm, and attentive in the dome, provides valuable contextualizing information for interpreting their physiological response pattern. Across the seven included interventions, EC01 exhibited decreased RMSSD in five sessions (daily average: 30.2 ms; intervention average: 26.1 ms) and increased HR in five sessions (daily average: 88.4 bpm; intervention average: 92.9 bpm). The RMSSD decrease combined with the HR increase suggests an overall excitatory response pattern to the intervention. Given EC01’s lack of physical exertion throughout the day, this pattern might reflect the increased cognitive engagement or emotional stimulation as a response to being in the dome and watching virtual nature-based videos. Since examining the time-series plots did not reveal apparent patterns, we concluded that EC01’s responses were session-specific and most likely tied to the cognitive engagement in the interventions.

#### 4.2.2 EC02

EC02 visited the dome 21 times for an average of 40.5 min, predominantly in the afternoon. Their profile was similar to EC01, as in being bedbound, generally calm, and attentive in the dome. Across the 14 included intervention sessions, EC02 exhibited a more mixed autonomic response as their RMSSD increased in eight sessions and decreased in six, while their HR increased in ten and decreased in four (daily average: 50.6 ms, 71.9 bpm; intervention average: 48.5 ms, 74.3 bpm). Examining the time-series data showed two distinct peaks in RMSSD (interventions 5–7 and 12–15), while HR generally trended downward across sessions. Considering EC02’s overall change in averages, the response pattern is similar to EC01, showing a slight decrease in RMSSD combined with an increase in HR. This finding once more might be indicative of cognitive engagement while watching virtual nature videos.

#### 4.2.3 EC03

While EC03 visited the dome 11 times for an average of 33 min per session, only two interventions were included in the analysis. The sessions generally took place during the morning and afternoon. Both interventions coincided with a decrease in RMSSD and an increase in HR across interventions (daily average: 47.6 ms, 79.0 bpm; intervention average: 40.4 ms, 81.2 bpm). Their profile, including bedbound, generally calm, and attentive in the dome, as well as the response pattern aligns with the suggested excitatory response pattern as found in EC01 and EC02. Finally, the time-series plots show a decrease in both RMSSD and HR over time, which might suggest a reduced novelty effect.

#### 4.2.4 EC04

EC04, who uses a wheelchair and was described by staff as generally very active and attentive in the dome, completed nine interventions (average session length: 38.5 min) of which three were included in the analysis. Sessions were conducted primarily in the morning and afternoon. While HR decreased across all three interventions relative to the daily average (daily average: 60.2 bpm; intervention average: 56.8 bpm), RMSSD indicated a variable pattern; with one intervention showing slight increases, whereas two sessions exhibited a pronounced decrease (daily average: 53.9 ms; intervention average: 47.1 ms). Longitudinally, RMSSD declined across interventions, potentially suggesting increasing cognitive engagement. Combined, the findings may point toward a mild calming effect considering the HR pattern, with increasing signs of cognitive engagement.

#### 4.2.5 EC05

EC05, who uses a wheelchair and was described by staff as attentive and occasionally fearful in the dome and throughout the day, visited the dome 11 times for an average of 43.5 min per session. The analysis included 10 sessions, mostly conducted in the afternoon, of which 8 showed a decrease in RMSSD and 6 an increase in HR (daily average: 18.7 ms, 96.9 bpm; intervention average: 14.6 ms, 97.5 bpm). The average values, both during the intervention and throughout the day, show a low baseline RMSSD and high baseline HR, indicative of a chronically activated sympathetic nervous system. While the interventions predominantly coincided with reduced parasympathetic activity, the direction in HR varied. Considering these findings in the context of EC05’s profile might suggest that they have mostly experienced the dome as exciting and cognitively engaging rather than calming.

#### 4.2.6 EC34

EC34, using no mobility device and described by staff as frequently agitated and restless, visited the dome 12 times (average session length: 29.5 min) of which 7 sessions, mostly in the afternoon, were included in the analysis. In contrast to the previous participants, EC34 exhibited a different response pattern with RMSSD decreasing in six and HR in five sessions (daily average: 35.8 ms, 105.3 bpm; intervention average: 20.4 ms, 94.0 bpm). This combination of lower parasympathetic activity paired with lower HR might be interpreted as general cognitive engagement with cooccurring cardiovascular recovery. Aligned with the participant profile and staff observations, the interventions seem to have had a calming effect on EC34’s generally high baseline values while keeping them cognitively engaged.

#### 4.2.7 EC36

EC36, similar to EC34 in their mobility and behavioral profile, visited the dome 8 times for an average of 29.5 min, mostly during the morning. Out of the three interventions included, all sessions exhibited consistent decreases in both RMSSD and HR (daily average: 22.5 ms, 77.3 bpm; intervention average: 18.0 ms, 75.1 bpm). This consistent pattern again suggests that participants in moments of agitation and restlessness may have benefited from the intervention’s calming effects by shifting their focus to the virtual nature videos, coinciding with a decrease in HR as well as RMSSD.

### 4.3 Interpretation of Findings in context

Taken together, the case-level analyses revealed a potential two-fold response pattern when examining the physiological changes in the context of the participants’ profiles and baseline levels of arousal. Participants described by staff as predominantly calm and bedbound (EC01-EC03) consistently showed signs that might be interpreted as increased cognitive and emotional engagement, as indicated by decreases in RMSSD combined with increases in HR. Given their otherwise low-stimulation daily routines and limited physical activity, this pattern likely reflects heightened stimulation, novelty, and cognitive engagement as a response to the immersive dome environment.

In contrast, participants characterized by staff as agitated, restless, or physically active (EC34, EC36, and EC04) exhibited decreases in RMSSD and HR, which may be indicative of cognitive engagement paired with cardiovascular recovery. For these individuals, the interventions appeared to have had a regulatory effect potentially explained by redirecting their attention and facilitating emotional and physiological calming during periods of elevated arousal.

Interpreted in light of the earlier-introduced attention restoration theory, these findings suggest that virtual nature exposure may operate by inducing effortless attention toward softly engaging stimuli, thereby supporting attentional regulation. In individuals with low baseline stimulation, this attentional shift increases cognitive activation, while for individuals in agitated or restless states the attentional shift reduces attentional overload and elicits calmness. The latter also aligns with the stress reduction theory, stating that nature exposure induces physiological relaxation in moments of elevated baseline arousal.

Some of these findings align with previous research in populations without disability, where exposure to real and virtual nature has been associated with reductions in HR and other stress-related physiological markers (Annerstedt et al., 2013; Kumpulainen et al., 2024). However, the consistent decrease in RMSSD observed across participants in this pilot study might suggest that the cognitive load associated with immersive virtual nature exposure may differ in this population. It remains speculative whether this response can be primarily attributed to the participants’ profiles, their daily routines at the care facility, the novelty of the intervention, or other factors altogether.

Overall, these findings underscore the importance of interpreting physiological changes not as simply positive or negative, but as meaningful reflections of individual profiles, baseline arousal, and experiences. Accordingly, virtual nature interventions may operate through different regulatory pathways depending on the needs and characteristics of each individual.

### 4.4 Implications for care facilities

The results of this pilot study provide preliminary evidence for the feasibility and potential benefits of implementing dome-based virtual nature interventions within institutional care settings.

For individuals with limited mobility and restricted access to natural environments, such dome-based interventions may offer an accessible, low-risk alternative to direct nature contact. Importantly, the active involvement and facilitation of the professional nursing staff emerged as a key enabling factor in this study and appears to be a prerequisite for a successful implementation. Supporting this, staff interviews conducted as part of the wider project and outside the scope of the present study suggested that staff responded overly positive to the dome environment (Niemi et al., unpublished manuscript). They indicated using it as a practical tool to calm residents down in moments of distress and expressed willingness to incorporate it into the regular routine. While these observations come from a different section of the wider project and should be interpreted cautiously, they tentatively support the feasibility of these dome-based interventions from an implementation perspective. From a healthcare perspective, virtual nature-based interventions may support stress regulation, promote emotional wellbeing, and contribute to the enrichment of daily routines. The intervention can be easily tailored to individual preferences by adjusting the timing, duration, and content, thereby making it an adaptable tool within care settings. Moreover, as participation in the VR dome experience is always voluntary and guided by care personnel’s interpretation of participants’ preferences, this type of intervention may also strengthen residents’ sense of autonomy and self-determination.

Finally, this study’s dome-based format differs significantly from head-mounted VR glasses, which to this point are more commonly used in research. Dome environments offer a shared and less physically intrusive alternative that may be better suited for individuals with intellectual and/or physical disabilities, as they do not require participants to wear equipment or tolerate close contact with a device.

### 4.5 Limitations and future directions

There are several limitations to this pilot study that warrant caution when interpreting its findings and generalizability. First, the small sample size (*N* = 7) limits generalizability to broader populations with intellectual and/or physical disability. Second, technical issues with the timestamping mechanism of the Firstbeat devices led to the exclusion of 3 recording sessions, thereby impacting consistency across cases and their interpretability. Third, while additional contextual information such as medical history, medications, and comorbidities would have significantly improved our interpretative precision, the absence of detailed clinical information was a deliberate choice to protect the participants’ identity given our small sample size. We acknowledge that this decision limits the depth of our physiological interpretation and represents one of this study’s main limitations.

Fourth, growing evidence suggests that the circadian rhythm significantly impacts the autonomic nervous system (Niwa et al., 2011; Shen et al., 2025). Despite some research finding altered circadian impacts in clinical populations (e.g., Parkinson’s disease; Niwa et al., 2011), we decided against accounting for the circadian rhythm as our participants lacked a uniform diagnostic basis. Hence, we acknowledge that the current study design cannot fully distinguish between intervention and time-of-day effects, as session timing varied across participants and days.

Fifth, the sole reliance on physiological data represents a broader methodological limitation. The absence of additional contextual data sources, including structured observation logs, participant reports, sleeping and activity logs, hindered our interpretation from being more than speculative. Incorporating such data sources in future research would enable more grounded interpretations of the observed physiological changes. While staff interviews were conducted as part of the broader project and informed our interpretation to some degree, they were not systematically collected in accordance with this study’s protocol, thereby limiting the interpretative weight.

Considering these limitations, future research should therefore replicate this study with larger samples, enhanced data completeness, and richer contextual and qualitative assessment. This could comprise caregiver interviews, structured observation protocols, and where possible participant self-report. In addition, we recommend comparing virtual versus real outdoor nature exposure to examine whether both modalities yield similar physiological responses and benefits.

## 5 Conclusion

This pilot study provides preliminary evidence on the feasibility and potential benefits of tailored virtual nature interventions for individuals with intellectual and/or physical disability. To our knowledge, this is among the first studies to examine physiological responses to virtual nature within this population.

Consistent with our hypothesis, the findings suggested measurable changes in participants’ HRV and HR, and these responses appeared to be participant dependent. While some individuals showed physiological patterns indicative of calming or stress-reducing effects, others displayed patterns that may reflect excitement and heightened cognitive engagement. This two-fold response pattern appeared to align with the participants’ profiles, such that restless and agitated participants exhibited a calming response, whereas already calm residents experienced the dome as stimulating.

Taken together, while preliminary, these findings point to the potential of tailored virtual nature interventions as a scalable, accessible, and adaptable tool for promoting well-being in institutional care facilities. This study lays important groundwork by demonstrating the potential of virtual nature in care settings and emphasizing the need for an individualized understanding of how heterogeneous groups respond to virtual nature environments.

## Data Availability

The raw data supporting the conclusions of this article will be made available by the authors, without undue reservation.
